# Exosomal MFI2-AS1 sponge miR-107 promotes non-small cell lung cancer progression through NFAT5

**DOI:** 10.1186/s12935-023-02886-x

**Published:** 2023-03-18

**Authors:** Jingwei Xu, Hui Wang, Baoyu Shi, Ning Li, Guopeng Xu, Xiaopei Yan, Li Xu

**Affiliations:** 1 Department of Cardiothoracic Surgery, The Affiliated Suzhou Hospital of Nanjing Medical University, Suzhou Municipal Hospital, Suzhou, 215002 China; 2 Department of Pulmonary and Critical Care Medicine, The Affiliated Suzhou Hospital of Nanjing Medical University, Suzhou Municipal Hospital, 26 Daoqian Street, Gusu District, Suzhou, 215002 China

**Keywords:** Non-small cell lung cancer, MFI2-AS1, miR-107, NFAT5, Pre-metastatic niche formation

## Abstract

**Background:**

Non-small cell lung cancer is a heterogeneous disease driven by extensive molecular alterations. Exosomes are small vesicles with diameters ranging from 30 to 150 nm released by various cell types and are important mediators of information transmission in tumor cells. Exosomes contain proteins, lipids, and various types of nucleic acids, including miRNAs and even DNA and RNA. MFI2 Antisense RNA 1 (MFI2-AS1) is a long noncoding RNA known to promote cell proliferation, metastasis and invasion in a variety of malignancies.

**Methods:**

The relative expression of MFI2-AS1 in NSCLC tissues was examined using RNA fluorescence in situ hybridization (FISH) staining. Transwell migration and wound healing assays were used to analyze cell migration and invasion abilities. Tube formation is used to assess angiogenic capacity. CCK8 was used to assess cell proliferation ability. RNA immunoprecipitation (RIP) experiments confirmed that MFI2-AS1 acts as a competing endogenous RNA (ceRNA) for miR-107. Dual-luciferase reporter assays were used to identify potential binding between MFI2-miRNA and target mRNA. In vivo experiments were performed by injecting exosomes into subcutaneous tumors to establish animal models.

**Result:**

Exosomal MFI2-AS1 increases NFAT5 expression by sponging miR-107, which in turn activates the PI3K/AKT pathway. We found that the MFI2-AS1/miR-107/NFAT5 axis plays an important role in exosome-mediated NSCLC progression, is involved in pre-metastatic niche formation, and can be used as a blood-based biomarker for NSCLC metastasis.

**Conclusion:**

We demonstrate that MFI2-AS1 is upregulated in exosomes secreted by metastatic NSCLC cells and can be transferred to HUVECs, promoting angiogenesis and migration.

**Supplementary Information:**

The online version contains supplementary material available at 10.1186/s12935-023-02886-x.

## Introduction

Non-small cell lung cancer (NSCLC) accounts for 85% of lung cancer cases and is a disease with high morbidity and mortality [1, 2]. Because early-stage NSCLC has no obvious symptoms, most patients are at an advanced stage at the time of diagnosis, losing the opportunity to surgically remove the tumor, and the 5 year survival rate is as low as about 24% [3]. Angiogenesis involves several complex steps in tumor growth, invasion and metastasis and plays an important role in NSCLC and most malignancies [4]. In addition, anti-angiogenic therapy has been shown to effectively prevent tumor growth and metastasis [5, 6].

Exosomes are extracellular vesicles with an average diameter of 30–150 nm, which originate from endocytic membranes and serve as vehicles for intercellular communication with the same topology as cells, remodeling the extracellular environment or transmitting signals and molecules to adjacent recipient cells [7–9]. Exosomes are cancer-derived factors that form a pre-metastatic niche and initiate distant metastasis [10–13]. Differences in exosome function between diseased and healthy individuals have attracted much attention from researchers due to their potential use in numerous pathological and physiological processes in various malignancies [14].

LncRNAs are non-coding RNAs with a length of more than 200 nucleotides and have no obvious protein-coding capacity [15, 16]. Accumulating evidence indicates that many lncRNAs are highly expressed in tumor cells, packaged into exosomes, and transported to the corresponding recipient cells, thereby playing an important role in intercellular communication. Exosome-derived lncRNAs affect tumor growth, metastasis, invasion, and prognosis by regulating the tumor microenvironment [17]. For example, hypoxic tumor-derived exosomal long non-coding RNA UCA1 promotes pancreatic cancer angiogenesis, which in turn leads to its metastasis Non-coding RNA MFI2-AS1 is highly expressed in various tumor cells [18]. For example, the non-coding RNA MFI2-AS1 is highly expressed in colorectal cancer and promotes colorectal cancer cell proliferation, migration and invasion through the miR-574-5p/MYCBP axis [19]. LncRNA MFI2-AS1 promotes HCC progression and metastasis by upregulating FOXM1 expression as a competing endogenous RNA for miR-134 [20]. In addition, the lncRNA MFI2-AS1/miR-125a-5p axis up-regulated TRIAP1 to promote thyroid cancer tumorigenesis [21]. However, the corresponding study of LncRNA MFI2-AS1 in NSCLC is lacking.

## Methods

### Specimens of a clinical nature

Histologically confirmed NSCLC tissue and serum samples (10 with metastasis and 10 without metastasis) were obtained from 20 patients who underwent surgery in Suzhou Municipal Hospital. 20 healthy serum samples matched the gender and age of the above patients were obtained from the Department of Respiratory Medicine, Suzhou Municipal Hospital. All clinical specimens were stored at − 80 °C after collection and frozen in liquid nitrogen. This study was approved by the Ethics Committee of Suzhou Hospital Affiliated to Nanjing Medical University, and all participants signed written consent.

### Cell culture

Human normal bronchial epithelial cells (16HBE) and NSCLC cell lines PC9, A549 and H1299 were purchased from the American Type Culture Collection (ATCC). Cells were cultured in RPMI 1640 medium supplemented with 1% penicillin/streptomycin, 10% fetal bovine serum (FBS) in a 37 °C and 5% CO2 incubator. All media and reagents were purchased from Gibco, USA.

### Exosomes isolation and characterization

Exosomes were extracted from NSCLC patient serum and NSCLC culture medium by ultracentrifugation. After the cells reached about 60% confluency, the medium was changed to RPMI 1640 medium containing 10% exosome-depleted FBS, and the cell supernatant was collected 2 days later and centrifuged at 500 g/16,800 g, respectively, at 4 °C. 10/30 min. The supernatant was separated, passed through a 0.22 um filter (Millipore) and then ultracentrifuged at 110,000 g for 70 min at 4 °C. After washing with phosphate buffered saline (PBS), ultracentrifuge at 110,000 g for 70 min at 4 °C and resuspend in PBS. Exocrine quantification by BCA protein detection kit (KeyGEN BioTECH). Exosome morphology was observed using a Tecnai T20 transmission electron microscope (TEM) from FEI, and the size and number of exosomes were determined by Nano Sight NS 300 system (Nano Sight Technology, Malvern, UK). Fluorescent labeling of exosomes with Sigma PKH67 membrane dye (green). Stained exosomes were washed in 10 ml PBS, then collected by ultracentrifugation and resuspended in PBS. For cellular uptake of exosomes, 2 µg of exosomes were incubated with 2 × 10^5^ recipient cells for 48 h.

### Quantitative real time‐polymerase chain reaction (qRT‐PCR)

Total RNA was first extracted from exosomes and cells using Trizol reagent (Invitrogen). Then cDNA for miRNA was synthesized using the miDE TECT A Track RT kit (RiboBio, China), while lncRNA and mRNA, complementary cDNA was obtained using a reverse transcription system (Promega, Madison, WI, USA). lncRNA, mRNA and miRNA expression levels were measured in triplicate samples by using SYBR Green PCR Master Mix (Applied Bio systems, Carlsbad, CA) on an ABI 7900 PCR system (Applied Bio systems) according to the manufacturer’s instructions. U6 and ACTB was selected as a normalization control for miRNA, lncRNA and mRNA sum, respectively.

### FISH

The relative expression of MFI2-AS1 in NSCLC tissues was examined using RNA fluorescence in situ hybridization (FISH) staining. Paraffin sections were dewaxed and rehydrated with ethanol. Paraffin sections were digested with proteinase K, fixed in 4% paraformaldehyde, hybridized overnight with a digoxigenin-labeled MFI2-AS1 synthetic oligonucleotide probe, followed by anti-digoxigenin-labeled peroxidase (anti-DIG Horseradish peroxidase [HRP]) (Roche, Mannheim, Germany) was incubated for 2 h. Subsequently, the paraffin sections were washed with water for 10 min and counterstained with hematoxylin. Images were observed and taken by using a fluorescence microscope (Olympus).

### Plasmid construction and transfection

We construct plasmids to knock down or increase the expression of cellular genes. PC9/A549 cells were plated at a cell density of 70% dish confluence for transfection. Plasmids transfected with Lipofectamine 2000 (Invitrogen, Carlsbad, CA). Plasmids for MFI2-AS1, NFAT5, miR-107 inhibitor or small interfering RNA (si-RNA) of MFI2-AS1 and NFAT5 were constructed by Genechem (Shanghai, China).

### Dual luciferase reporter gene assay

The pmirGLO Dual-Luciferase miRNATarget expression vector was purchased from GenePharma (Shanghai, China), and the luciferase reporter plasmid was inserted into the wild-type WT-MFI2-AS1 and Mut-MFI2-AS1 3'UTR sequences. Firefly luciferase was used as the primary reporter gene to regulate mRNA expression, and Renilla luciferase was used as a normalization control. Dual-luciferase reporter gene assay system (Promega) was used 48 h after transfection. The mean luciferase intensity was normalized to Renilla luciferase. Data are shown as mean ± SD and each experiment was performed three times.

### Western blot

Cells were harvested after 24 h of co-culture of exosomes with cells. After RIPA cleavage, total protein was extracted and assayed by BCA method. Quantitative denatured proteins (20 μg protein per lane) were separated on 10% SDS-PAGE and transferred to protein electrophoresis membranes, which were blocked with fast sealing solution. Follow the procedure for the first and second incubations. The expression of proteins is represented by grayscale values. List of primary antibodies: CD63 (ab134045, Abcam), Calnexin (ab133615, bacam), Tsg101 (ab125011, Abcam), NFAT5 (ab3446, Abcam), aAKT/p-AKT (CST, #4060, GAPDH (ab8245, Abcam).

### CCK8

We used Cell Counting Kit-8 (CCK8) to observe cell proliferation. The co-cultured cells were seeded into 96-well plates at 1000 cells/well, with 3 replicate wells per group. 2 h before observation, 10 μL of CCK8 reagent was added to each well for treatment. Finally, use absorbance at 450 nm in the dark to determine cell proliferation viability. Each sample were performed in three times.

### Tube formation

250 μL of Matrigel (BD Bioscience, USA) was added to a 48-well plate and resuspended at approximately 2 × 10^4^ cells per well. Tube formation was observed with an inverted microscope (Olympus Corporation, Tokyo, Japan) after 8–12 h of incubation. Quantify the branching of the tube structure according to the manufacturer's instructions after taking the images. Use Image J to measure and calculate the total length of tube formation in each well. Each sample was replicated three times.

### Wound-healing assay

Cells were resuspended and plated in serum-free medium and replated in 6-well plates to grow to 100% confluency. After scraping the cell monolayer vertically with a sterile pipette tip, wash the cells 3 times with 1% PBS. Wound fusion was observed under an inverted microscope (Olympus Corporation, Tokyo, Japan) and photographed at 0 and 36 h. Each sample was replicated three times.

### Transwell

Transwell migration assays used 8.0 μm Transwell permeable supports (Corning, New York, USA). Briefly, 4 × 10^4^ cells in 400 μL of serum-free medium were added to the upper chamber. Add 600 µL of medium containing 10% FBS to the lower chamber. The chambers were incubated in 5% CO2 at 37 °C for 24 h and fixed with paraformaldehyde for 30 min. After staining with 0.4% crystal violet for 15 min, cells were photographed and counted in 5 random fields. Each sample was replicated three times.

### RIP

We transfected HUVECs with pcDNA3.1-MFI2-AS1 or control for 48 h, then resuspended cells in radioimmunoprecipitation assay (RIPA) buffer and incubated for 30 min. After centrifugation, human anti-Ago2 antibody (Proteintech, USA) was added to the lysates and incubated for 4 h, selecting normal mouse immunoglobulin G as a negative control. We then resuspended the beads in RIPA buffer and treated the samples with proteinase K for 45 min in a 45 °C incubator. Trizol extracted immunoprecipitated RNA samples were tested by quantitative real-time PCR analysis.

### Animal experiment

5 × 10^6^ A549 cells were injected subcutaneously into the armpits of 4-week-old female nude mice. 20 mg of exosomes were injected into the right axillary tumor center of mice every 3 days. After 30 days, the mice were sacrificed, the subcutaneous tumors were removed, and the volume and weight were recorded. Afterwards, tumors were excised for immunohistochemical staining for CD34. All animal experiments were approved by the Animal Research Ethics Committee of Nanjing Medical University.

### Statistical analysis

SPSS19 Statistics software was used for statistical data analysis. Data are presented as mean ± standard deviation (SD) and each experiment was performed three times.

Student’s t-test or one-way analysis of variance (ANOVA) was used to compare statistics between two or more groups. P < 0.05 was considered statistically significant.

## Results

### LncRNA MFI2-AS1 is highly expressed in exosomes of NSCLC patients

**Fig. 1:**
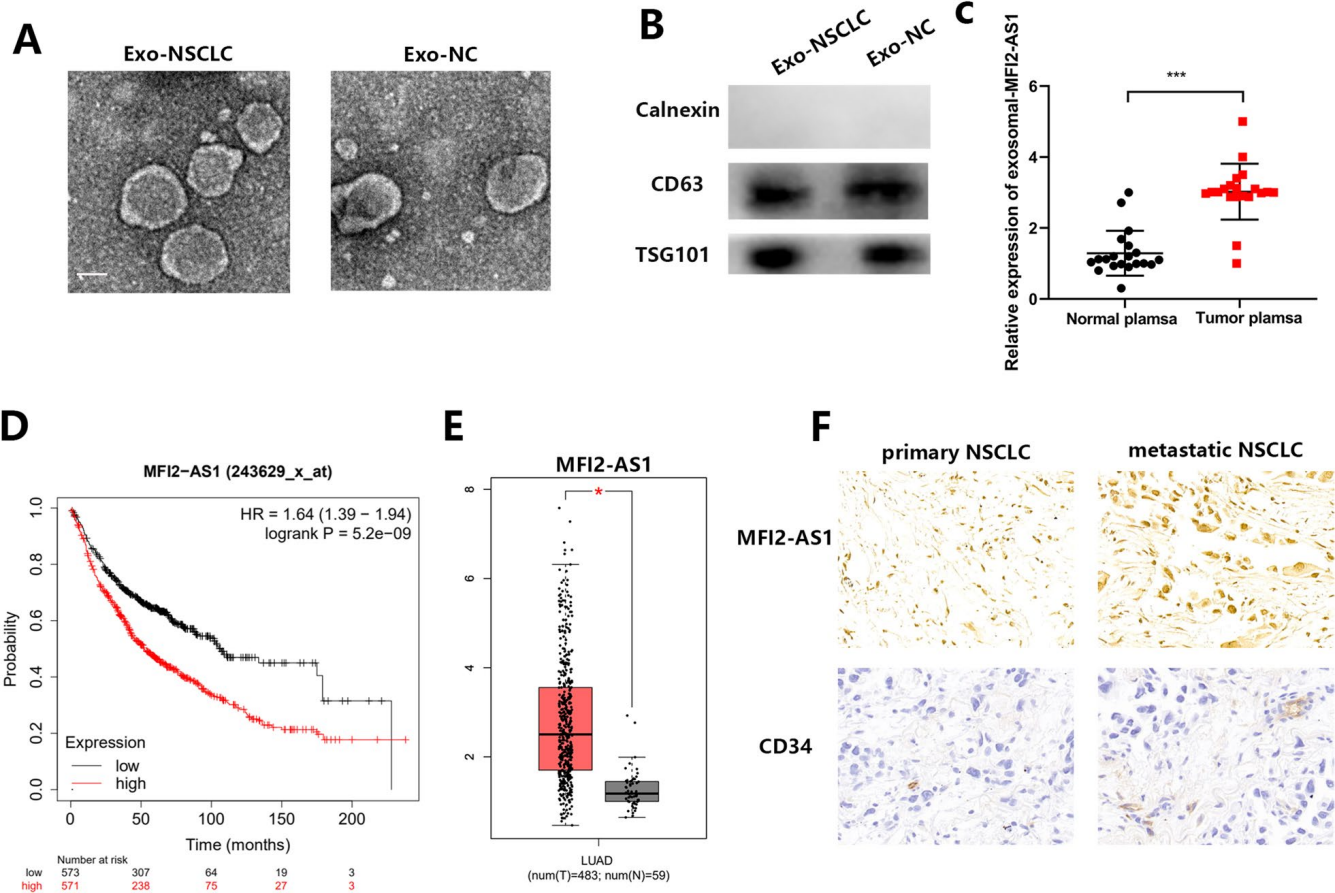
LncRNA MFI2-AS1 is highly expressed in exosomes of NSCLC patients. **A** TEM image of exosomes; Scale bar, 50 μm. **B** Western blot analysis of exosomal markers CD63, Calnexin and TSG101. **C** Expression levels of MFI2-AS1 in plasma exosomes from NSCLC patients (20 normal, 20 tumor). **D** Association of MFI2-AS1 with survival in NSCLC patients. **E** Expression levels of MFI2-AS1 in NSCLC patient tissues. **F** Representative images of ISH staining for MFI2-AS1 and immunohistochemical (IHC) staining for CD34 in NSCLC tissue specimens. The data expressed as the mean ± SD. (*P < .05; **P < .01; ***P < .001)

To verify the presence of exosomal LncRNA MFI2-AS1. First we extracted exosomes from the plasma of NSCLC patients (Exo-NSCLC) or healthy controls (Exo-NC), respectively. The morphology of exosomes was examined by TEM (Fig. 1A). Furthermore, western blotting showed that the vesicles were positive for the exosomal markers CD63 and TSG101, but negative for Calnexin (Fig. 1B). The results of qRT-PCR showed that the expression of LncRNA MFI2-AS1 in exosomes in the serum of NSCLC patients was higher compared with that of healthy patients (Fig. 1C). Through survival curve analysis, we found that patients with high expression of LncRNA MFI2-AS1 had poor prognosis (http://kmplot.com/analysis/) (Fig. 1D). We found in GEPIA (http://gepia.cancer-pku.cn/) that the expression of LncRNA MFI2-AS1 was also higher in LUAD tissues than in normal tissues (Fig. 1E). More interestingly, by IHC we found that LncRNA MFI2-AS1 and CD34 were significantly elevated in NSCLC metastatic patient tissues compared to non-metastatic patient tissues (Fig. 1F). So we envisioned whether the non-coding RNA MFI2-AS1 promoted the metastasis of NSCLC through exosome transfer to vascular endothelial cells.

### MFI2-AS1 is highly expressed in NSCLC and can be transferred to HUVECs via exosomes

**Fig. 2:**
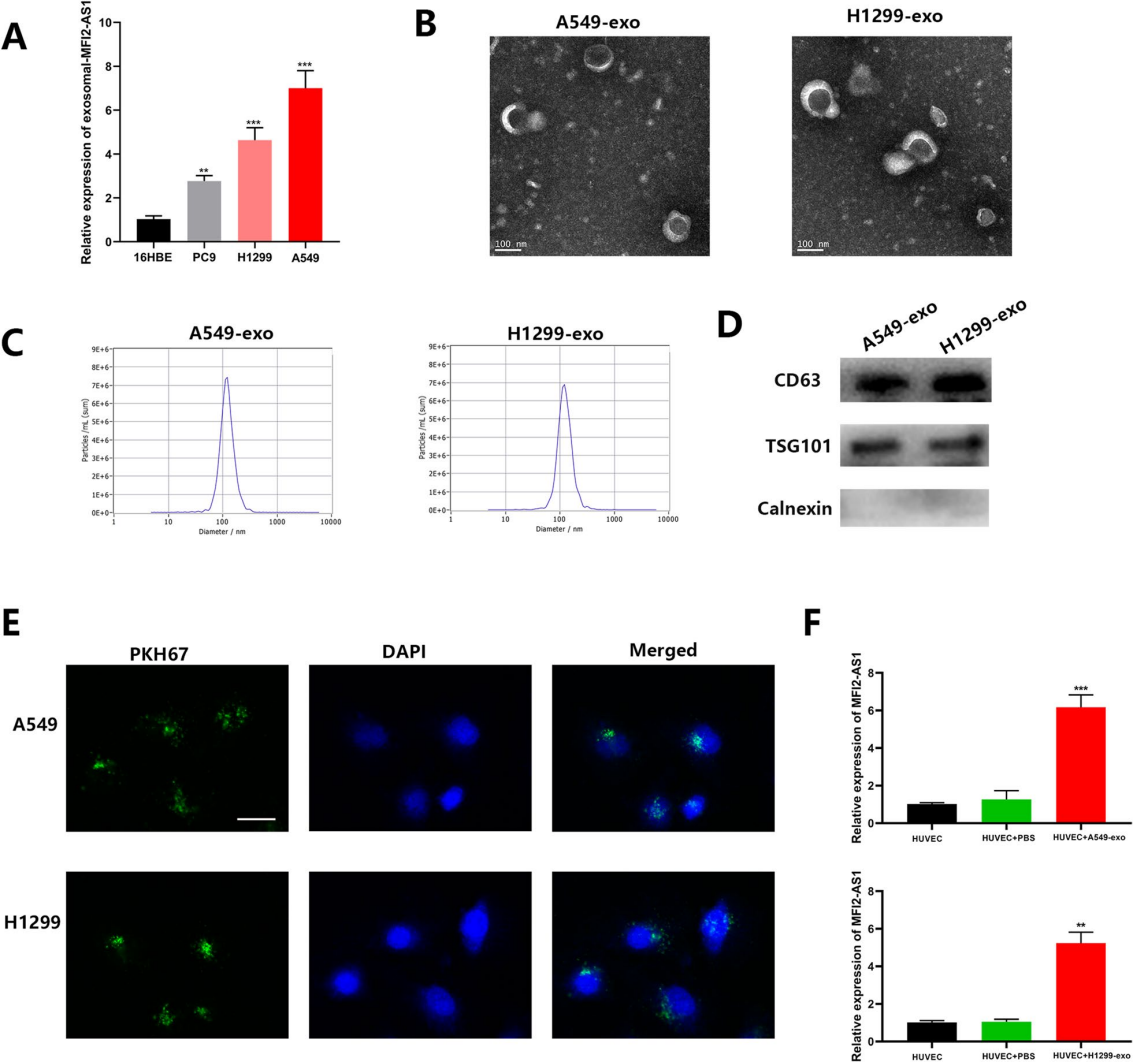
MFI2-AS1 is highly expressed in NSCLC and can be transferred to HUVECs via exosomes. **A** Detection of MFI2-AS1 levels in NSCLC cell lines by qRT-PCR. **B** TEM image of exosomes. **C** Nano Sight NS 300 system analysis confirmed the particle size distribution between 30 and 150 nm. **D** Western blot analysis of exosomal markers CD63, Calnexin and TSG101. **E** PKH67 staining to determine internalization of exosomes in recipient cells. Scale bar, 20 μm. **F** The level of MFI2-AS1 in recipient cells was detected by qRT-PCR. The data expressed as the mean ± SD. (*P < .05; **P < .01; ***P < .001)

We first measured MFI2-AS1 expression in cell lines and found that MFI2-AS1 expression was higher in NSCLC cells than in normal bronchial epithelial cells (Fig. 2A). We first extracted and purified exosomes from conditioned media and from two cell lines (H1299 and A549). Their cup-shaped morphology as well as size and number of exosomes were determined by TEM analysis and Nano Sight particle tracking analysis, respectively (Fig. 2B–C). In addition, the exosomal markers TSG101, CD63 and Calnexin were identified using western blot analysis (Fig. 2D). To confirm the manner in which MFI2-AS1 is delivered between cells, we performed co-culture experiments to determine whether exosomes and their contents could be internalized by target cells. We first stained exosomes with PKH67. After 24 h, we found that the stained exosomes rapidly entered HUVEC, mainly distributed around the nucleus (Fig. 2E). Through PCR experiments, we found that the expression of MFI2-AS1 increased after HUVEC ingested exosomes (Fig. 2F).

### Exosomal MFI2-AS1 promotes migration and tube formation of HUVECs in vitro

**Fig. 3:**
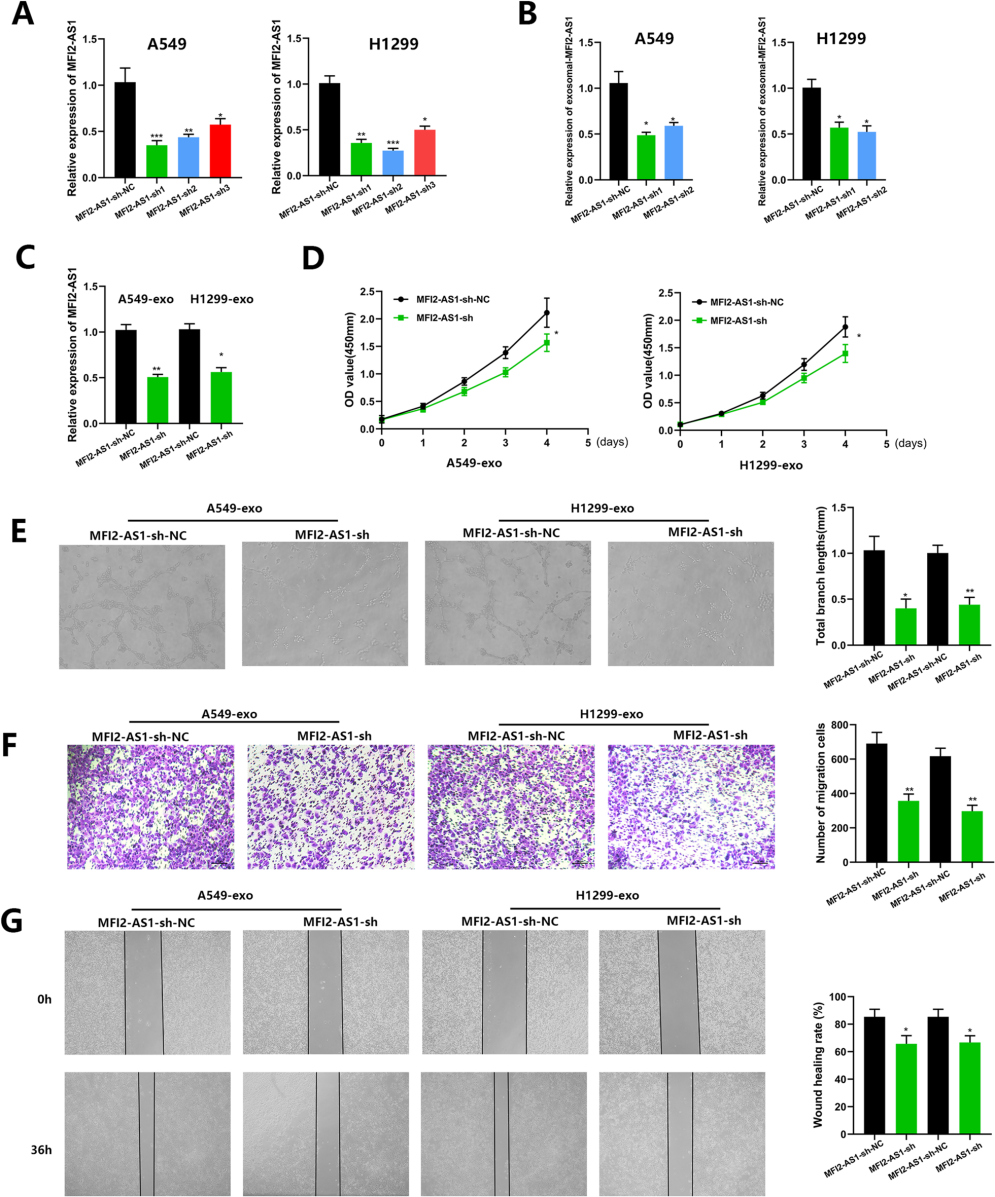
Exosomal MFI2-AS1 promotes migration and tube formation of HUVECs in vitro. **A**–**B** Real-time PCR analysis of MFI2-AS1 in NSCLC cells and exosomes after transfection of MFI2-AS1 shRNAs. **C** Exosomes isolated from NSCLC cells transfected with MFI2-AS1 shRNAs were pretreated with equal amounts of HUVECs for 24 h. **D** The proliferative capacity of HUVECs was assessed by CCK8 assay. **E** The tube-forming ability of HUVECs was assessed by the in vitro Matrigel tube-forming assay. **F**–**G** The cell migration ability of HUVECs was assessed by Transwell migration assay and wound healing. The data expressed as the mean ± SD. (*P < .05; **P < .01; ***P < .001)

We hypothesized that NSCLC-secreting exosomes could mediate a novel mechanism of intercellular communication between NSCLC and HUVECs through the delivery of MFI2-AS1, involved in metastasis and angiogenesis to promote NSCLC malignant progression. To confirm our conjecture, we performed functional experiments on co-cultured cells. We first inhibited the expression of MFI2-AS1 in A549 and H1299 cells by MFI2-AS1 short hairpin (sh) RNA, and verified the knockdown efficiency of MFI2-AS1 in cells and cell supernatant exosomes by PCR (Fig. 3A, B). Exosomes isolated from MFI2-AS1-suppressed NSCLC were then co-cultured with HUVECs to determine whether exosomal MFI2-AS1 mediates angiogenesis and migration of HUVECs. After 24 h of co-culture, we verified the expression of MFI2-AS1 in HUVECS cells by PCR (Fig. 3C). The proliferation and angiogenesis capacity of HUVECs were measured by CCK8 assay and tube formation assay, and it was found that MFI2-AS1-sh-Exos, compared with MFI2-AS1-sh-NC-Exos, MFI2-AS1-sh-Exos significantly reduced HUVEC proliferation and angiogenesis (Fig. 3D, E). Similarly, transwell and Wound-¬healing assay were used to measure the migration ability of HUVECs. Compared with MFI2-AS1-sh-NC-Exos, MFI2-AS1-sh-Exos significantly reduced the migration efficiency of HUVEC (Fig. 3F, G).

### MFI2-AS1 as a competing endogenous RNA (ceRNA) for miR-107 in HUVECs

**Fig. 4:**
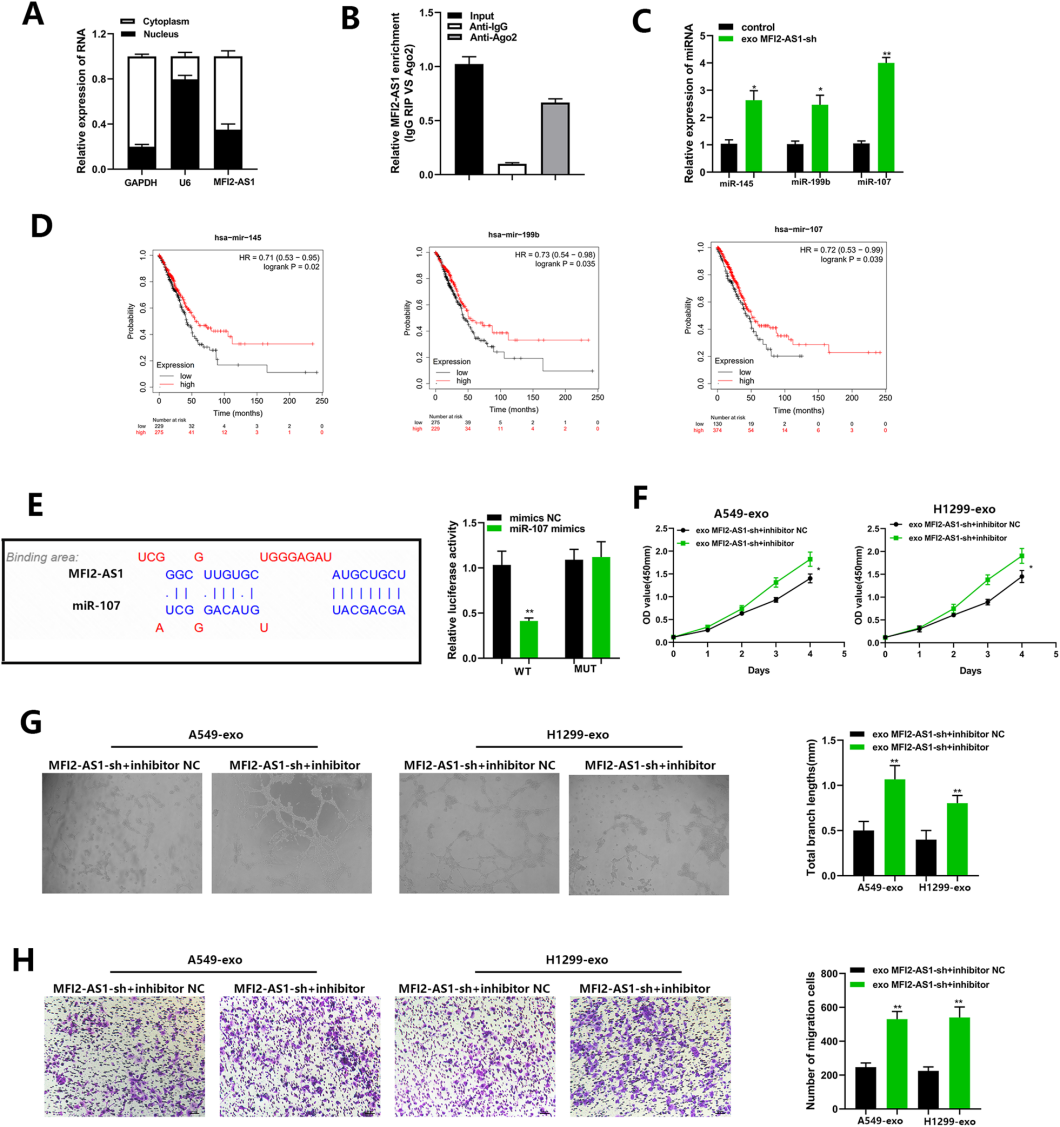
MFI2-AS1 as a competing endogenous RNA (ceRNA) for miR-107 in HUVECs. **A** After nuclear/cytoplasmic separation, MFI2-AS1 is mainly located in the cytoplasmic fraction. U6 and glyceraldehyde 3-phosphate dehydrogenase (GAPDH) were used as controls. **B** RIP assay was used and qRT-PCR was performed on co-precipitated RNA. Compared with IgG immunoprecipitation, RNA levels were fold enriched in Ago2. **C** Real-time PCR analysis of 3 candidate miRNAs after co-culture of HUVECs with exo-MFI2-AS1-shRNA. **D** Association of NSCLC patient survival among 3 candidate miRNAs. **E** Sequence alignment display and dual-luciferase reporter of the binding site of miR-107 to the MFI2-AS1 region. **F** The proliferative capacity of HUVECs was assessed by CCK8 assay. **G** The angiogenic capacity of HUVECs was assessed by in vitro Matrigel tube formation assay. **H** The cell migration ability of HUVECs was assessed by Transwell migration assay. The data expressed as the mean ± SD. (*P < .05; **P < .01; ***P < .001)

To explore the specific mechanisms of MFI2-AS1-driven angiogenesis and HUVEC migration, we first determined the localization of MFI2-AS1 in HUVECs. In FISH experiments, after nuclear/cytoplasmic separation of HUVECs, we found that MFI2-AS1 was mainly localized to the cytoplasmic fraction (Fig. 4A). We hypothesized that it may function as a ceRNA in angiogenesis and vascular endothelial cell migration in NSCLC. To test this, we performed RNA immunoprecipitation (RIP) assays on Argonaute2 (Ago2), a core component of the RNA-induced mediator complex. The experimental results showed that the enrichment of MFI2-AS1 on Ago2 was increased in HUVECs (Fig. 4B). These results suggest that MFI2-AS1 acts as a ceRNA to reduce the expression of its downstream proteins by competitively binding to specific miRNAs. We predicted 10 miRNAs involved in MFI2-AS1 sponging by the bioinformatics tool miRcode (http://www.mircode.org/) (Additional file 1: Figure S1A). By survival prognostic analysis, three candidate miRNAs met the criteria (http://kmplot.com/analysis/) (Fig. 4D). We found that miR-107 was most significantly upregulated in exo-MFI2-AS1-sh HUVECs by quantitative real-time PCR (Fig. 4C), indicating that miR-107 was the most suitable candidate for further analysis. The potential binding site of miR-107 in MFI2-AS1 was predicted by bioinformatics analysis(http://www.mircode.org/). Furthermore, luciferase reporter assays showed that miR-107 inhibited the luciferase activity of WT MFI2-AS1, but not mutant MFI2-AS1 (Fig. 4E). We next identified MFI2-AS1 as a competing endogenous RNA (ceRNA) for miR-107 in HUVECs by rescue experiments. Reduced restoration of miR-107 inhibited MFI2-AS1-mediated pro-angiogenic and migration (Fig. 4F–H).

### MFI2-AS1 promotes angiogenesis by regulating miR-107/NFAT5/AKT signaling pathway in HUVECs

**Fig. 5:**
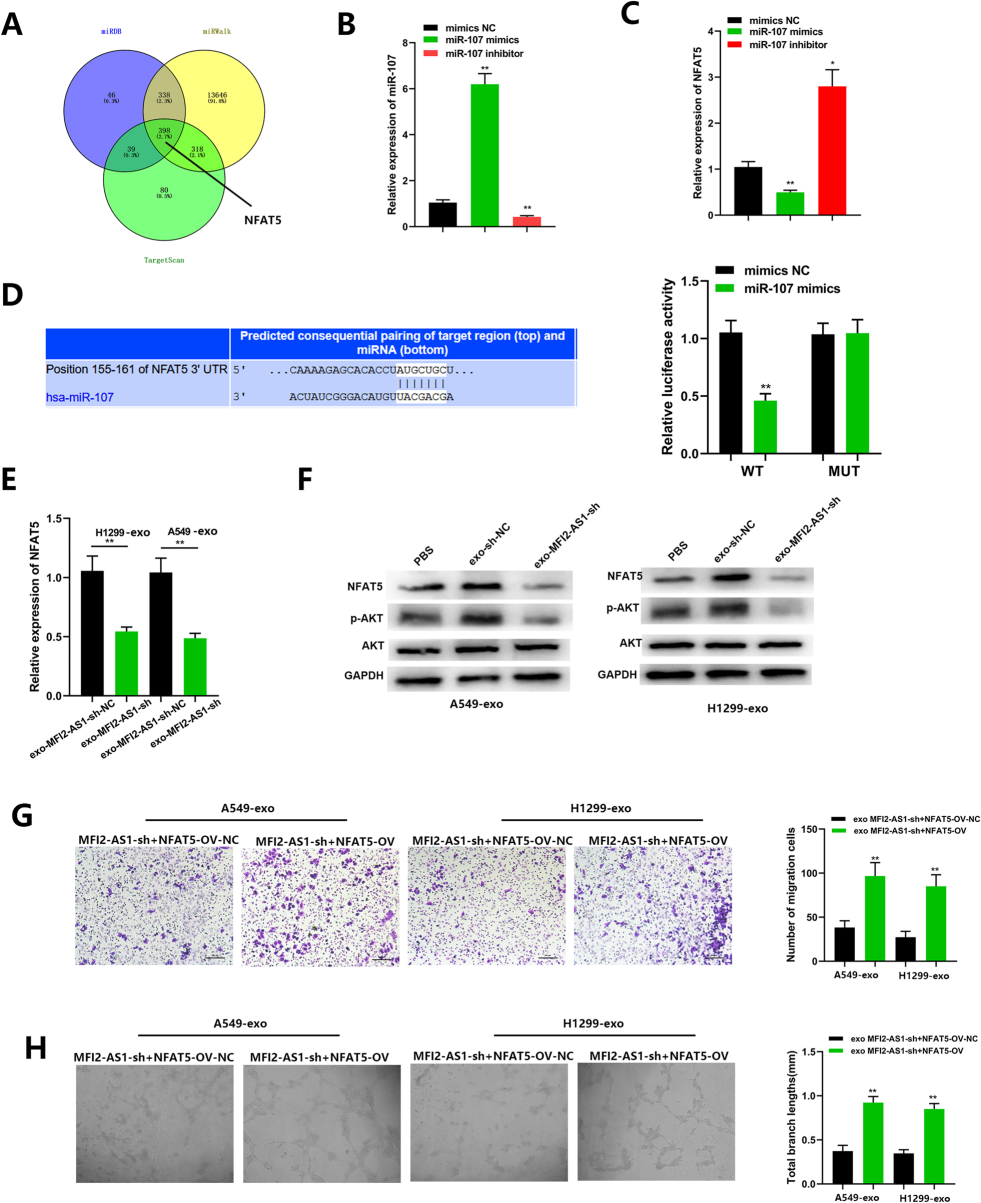
MFI2-AS1 promotes angiogenesis by regulating miR-107/NFAT5/AKT signaling pathway in HUVECs. **A** NFAT5 was discovered as a potential downstream target of miR-107 by using TargetScan, miRWALK and miRDB databases. **B** Real-time PCR analysis of miR-107 in HUVECs after transfection of miR-107 mimics and miR-107 inhibutor. **C** Real-time PCR analysis of NFAT5 in HUVECs after transfection of miR-107 mimics and miR-107 inhibutor. **D** Sequence alignment display of the binding site of miR-107 to the NFAT5 region and dual luciferase reporter. **E** Real-time PCR analysis of NFAT5 after co-culture of HUVECs with exo-MFI2-AS1-shRNA. **F** Western blot analysis of indicated proteins in exo-MFI2-AS1-sh HUVECs. **G**–**H** The effects of exo-MFI2-AS1 and NFAT5 on migration and angiogenesis were assessed by Transwell and angiogenesis assays. The data expressed as the mean ± SD. (*P < .05; **P < .01; ***P < .001)

To identify the genes that MFI2-AS regulates through miR-107 sponging, we first predicted potential target genes of miR-107. NFAT5 was discovered as a potential downstream target of miR-107 by using TargetScan, miRWALK, and miRDB databases (Fig. 5A). In order to investigate potential targets of miR-107, we predicted candidate gene targets by cross-output of three different prediction algorithms. We focused on factors that are up-regulated in NSCLC. Among them, by referring to the articles, we found that many candidate factors were low-expressed in NSCLC, which made no logical sense. In addition, there are many non-functional target candidates. That screened out a lot of potential targets. Some studies have reported that NFAT5 is up-regulated in NSCLC and other tumors, which is also very consistent with our study. Bioinformatics analysis showed that the binding score of 3 '-UTR of NFAT5 and miR-107 was higher. We then measured the expression levels of NFAT5 in miR-107 mimics and miR-107 inhibitor-transfected HUVECs by quantitative real-time PCR. We found that the mRNA level of NFAT5 was up-regulated in miR-107 inhibitor HUVECs, but down-regulated in miR-107 mimics HUVECs (Fig. 5B, C). To determine whether NFAT5 is a target of miR-107, we performed luciferase reporter experiments in HUVECs. As shown, the NFAT5 3'UTR contains a potential miR-107 binding site (https://www.targetscan.org/vert_72/). In addition, NFAT5-Wt and NFAT5-Mut were co-transfected into cells with miR-107 mimics or NC for dual-luciferase reporter assay, and compared with the NC group, NFAT5-Wt in the presence of miR-107 mimics The luciferase activity was inhibited (p < 0.05), suggesting that miR-107 could specifically bind to NFAT5 (Fig. 5D). Next, exosomes isolated from transfected NSCLC were co-cultured with HUVECs to explore the potential relationship between MFI2-AS1 and NFAT5. By PCR assay, we found that the mRNA level of NFAT5 was down-regulated in sh-MFI2-AS1 HUVECs compared with the control group (Fig. 5E). In addition, western blot analysis also indicated that the protein level of NFAT5 was also downregulated in sh-MFI2-AS1 HUVECs, and in addition sh-MFI2-AS1 could downregulate the phosphorylation level of AKT (p-AKT) (Fig. 5F). The AKT signaling pathway can regulate the function of vascular endothelial cells in HUVECs, further verifying our hypothesis. We next performed rescue experiments by transfection to increase NFAT5 levels in HUVECs. Restoration of overexpressed NFAT5 inhibited MFI2-AS1-mediated pro-angiogenic and migration (Fig. 5G, H).

### Exosomal MFI2-AS1 promotes angiogenesis in vivo

**Fig. 6:**
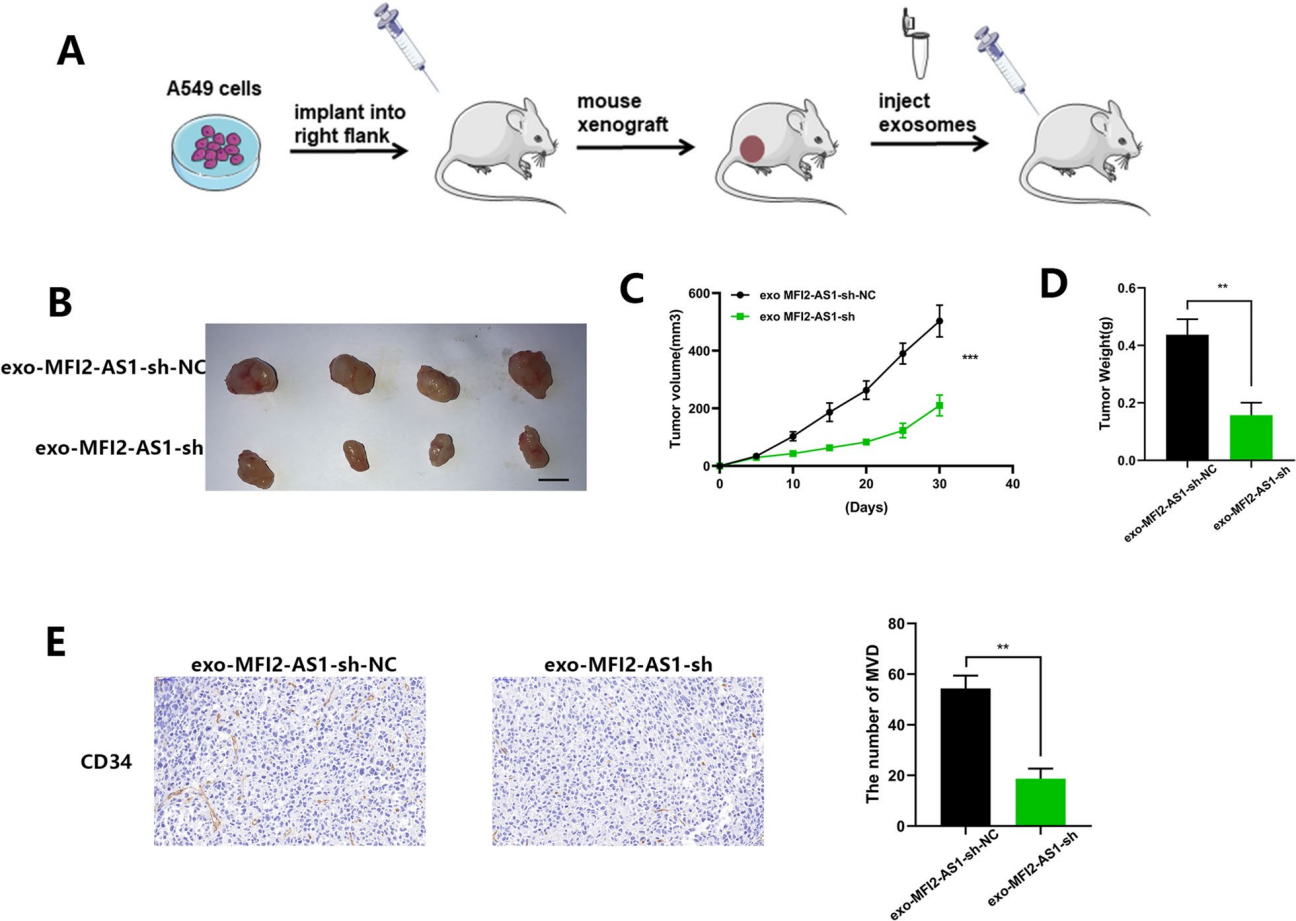
Exosomal MFI2-AS1 promotes angiogenesis in vivo. **A** Schematic flow chart of the mouse xenograft tumor model. **B** Images of excised tumors in xenografted mice (n = 4), the scale bar is 10 mm. **C** Tumor volume curve of each group. **D** Tumor weight in each group. **E** CD34 immunohistochemical staining to detect MVD in tumor tissue. The data expressed as the mean ± SD. (*P < .05; **P < .01; ***P < .001)

In vitro experiments have verified our earlier conjecture, and we further design in vivo experiments to confirm. We established a mouse xenograft tumor model to further determine the pro-angiogenic role of exosomal MFI2-AS1 in vivo (Fig. 6A). Compared with exo-MFI2-AS1-sh-NC treatment, exo-MFI2-AS1-sh-treated mouse xenografts reduced tumor volume (Fig. 6B–D). CD34 is a marker of microvascular formation, and we found by IHC staining that CD34 was decreased in the exo-MFI2-AS1-sh treated group compared with the control group (Fig. 6E). Therefore, we speculate that tumor-derived exosomal MFI2-AS1 promotes tumor growth and angiogenesis.

## Discussion

As in most solid tumors, the pre-metastatic niche is the favorable microenvironment created by the primary tumor for subsequent lymph nodes and organs, following the well-known “soil” and “seed” theory of metastasis [22–24]. According to the different histogenesis, EVs can be divided into microvesicles and exosomes, which play an important role in the remodeling of the tumor microenvironment. Their content includes nucleic acids, lipids, and proteins that can promote the malignant progression of cancer cells, among others [25]. Recent studies have shown that exosomes regulate the progression of malignant tumors by transferring multiple molecules, including CircNA, LncRNA, and miRNA, to recipient cells, acting as a guide for cell-to-cell communication. They have a wide range of uses in numerous pathological and physiological processes of various malignancies. Exosome-derived LncRNAs are involved in tumor proliferation and metastasis by regulating miRNAs or genes in target cells. We found that exo-MFI2-AS1 is involved in the formation of the pre-metastatic microenvironment in NSCLC and promotes the entry of exosomes into the peripheral blood circulation. NSCLC-derived exo-MFI2-AS1 plays critical roles in tumor formation, proliferation, invasion, metastasis and other processes. Therefore, we speculate that MFI2-AS1 may function as a prognostic oncogene in NSCLC and serve as a potential therapeutic target for NSCLC.

We first evaluated potential metastasis-associated LncRNAs in NSCLC. We found that the expression of MFI2-AS1 in serum exosomes of NSCLC patients was increased before surgery, and significantly decreased 1 week after surgery, indicating that exosomal MFI2-AS1 was derived from NSCLC. Previous studies have shown that MFI2-AS1 is closely associated with tumor recurrence and poor disease-free survival (DFS) [26]. Since MFI2-AS1 has been shown to play an oncogenic role in multiple cancers, we investigated whether exo-MFI2-AS1 could act as an oncogene in the pre-metastatic microenvironment of NSCLC. Functional experiments showed that the angiogenesis and invasion abilities of HUVEC cells were positively correlated with the content of MFI2-AS1 in cells, and reducing the expression level of MFI2-AS1 in cells could reduce the angiogenesis and invasion abilities of cells. These findings suggest that the metastasis-associated serum exosomal MFI2-AS1 is a novel oncogene in NSCLC and can be used as a potential metastasis biomarker and therapeutic target in NSCLC patients.

Next, we investigated the mechanism by which exo-MFI2-AS1 mediates HUVEC cell migration. FISH experiments confirmed that MFI2-AS1 was mainly located in the cytoplasm, and we speculated that it might play a role as a ceRNA in angiogenesis and vascular endothelial cell migration in NSCLC. miR-107 has a tumor suppressor effect and plays a role in inhibiting cancer development in a variety of cancers [27–30]. We confirmed that MFI2-AS1 acts as a competing endogenous RNA (ceRNA) for miR-107 in HUVECs by bioinformatics prediction, RIP, PCR, and dual-luciferase reporter assays, which we further verified by rescue experiments. Through three bioinformatics sites and dual luciferase reporter genes, we confirmed the target gene NFAT5 of miR-107. As an oncogene, NFAT5 is highly expressed in various tumors [31, 32]. Therefore, we further determined that MFI2-AS1 could promote HUVEC angiogenesis and metastasis by regulating NFAT5. During the course of this study, we observed that MFI2-AS1 acts as a competing endogenous RNA (ceRNA) for miR-107 in HUVECs, acting on NFAT5 to activate the AKT pathway. Therefore, exo-MFI2-AS1 promotes the formation of the pre-metastatic microenvironment in NSCLC and promotes tumor development.

Many recent studies have revealed the emerging biomarker roles of exosomes in cancer diagnosis and prognosis assessment. Exosomal integrins have been shown to be potential markers in certain organ-specific metastasis predictions. Exosomal miR-25-3p from colon cancer cells is involved in the formation of a pre-metastatic niche and can be used as a blood biomarker for colon cancer metastasis. Notably, it has been reported that lncRNA-MFI2-AS1, can serve as an independent prognostic indicator and potential therapeutic target for colon cancer. Here, our experiments showed that MFI2-AS1 levels in circulating exosomes from metastatic NSCLC patients were higher than those from patients without metastases, and the CD34 positivity rate in the tissues of metastatic patients was also significantly higher than that of non-metastatic patients. Collectively, we speculated that quantitative detection of MFI2-AS1 levels in circulating exosomes would be useful in diagnosing NSCLC metastasis and selecting patients at high risk of metastasis for preventive treatment.

## Conclusions

We demonstrate that MFI2-AS1 is upregulated in exosomes secreted by metastatic NSCLC cells and can be transferred to HUVECs, promoting angiogenesis and migration. Furthermore, NSCLC cell-derived exosomal MFI2-AS1 enhanced angiogenesis in vitro and in vivo by regulating the miR-107/NFAT5/AKT axis. In addition, exosomal MFI2-AS1 in human serum may serve as a new therapeutic target for the diagnosis of NSCLC metastasis biomarkers and the treatment of advanced NSCLC.

## Supplementary Information

**Additional file 1: Figure S1.** (A) 10 miRNAs involved in MFI2-AS1 sponging predicted by the bioinformatics tool miRcode (http://www.mircode.org/).

**Additional file 2.** miRDB.

**Additional file 3.** miRWalk.

**Additional file 4.** TargetScan.

## Data Availability

The datasets used and/or analyzed during the current study are available from the corresponding author on reasonable request.
