# Supplementary figures and images for: Exosomal MFI2-AS1 sponge miR-107 promotes non-small cell lung cancer progression through NFAT5
Source: Cancer Cell Int. 2023 Mar 18;23:51. doi: 10.1186/s12935-023-02886-x (PMC10024841; doi:10.1186/s12935-023-02886-x)

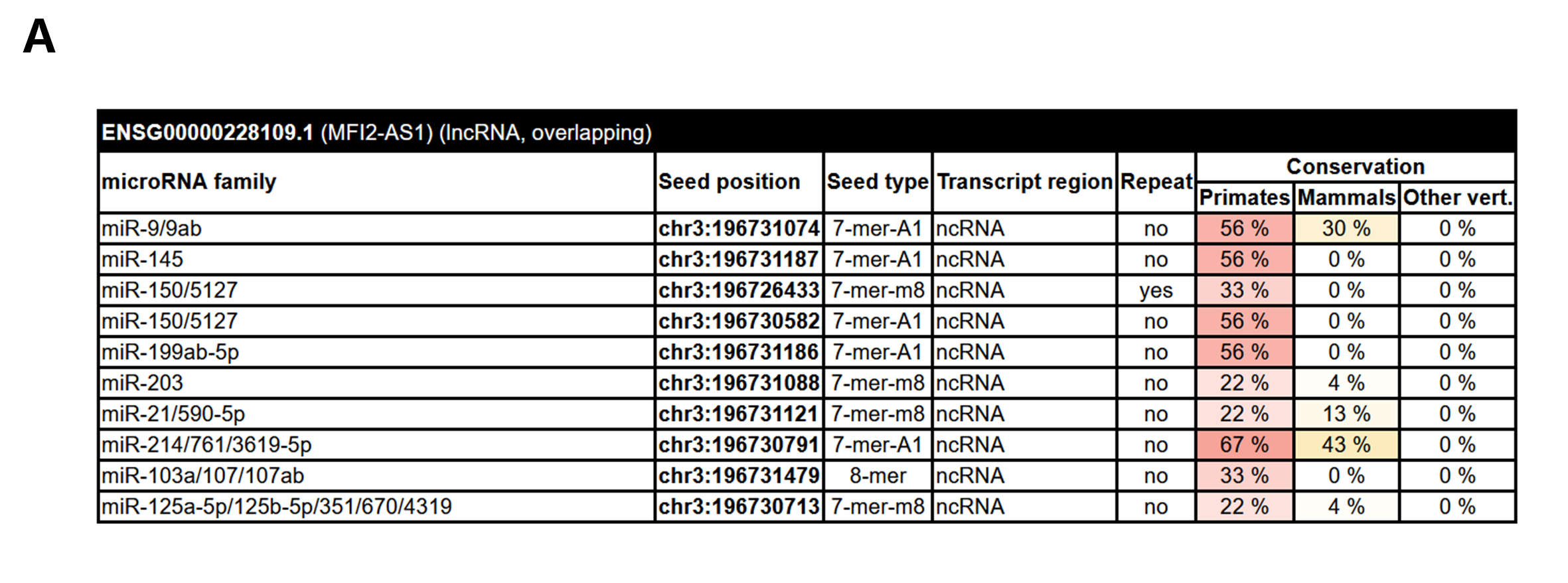

Supplement: Supplementary file 1 — Additional file 1: Figure S1. (A) 10 miRNAs involved in MFI2-AS1 sponging predicted by the bioinformatics tool miRcode (http://www.mircode.org/). [file 12935_2023_2886_MOESM1_ESM.tif]
